# Supplementary material for: Ectopic overexpression of Plasmodium falciparum DNA-/RNA-binding Alba proteins misregulates virulence gene homeostasis during asexual blood development
Source: Microbiol Spectr. 2025 Jan 27;13(3):e00885-24. doi: 10.1128/spectrum.00885-24 (PMC11878077; doi:10.1128/spectrum.00885-24)
Supplement: Supplemental material — Table S1; Figures S1 to S9 [file spectrum.00885-24-s0001.pdf]

Supplementary Material for *Acharya et al.* 2024

This document contains:

Supplementary Table S1

Supplementary Figures S1 to S9, with legends

The following are provided as Excel sheets:

Supplementary Table S2: Consolidated HTSeq-generated read counts for all *P. falciparum* genes in the transcriptomic datasets generated in this study

Supplementary Table S3: List of differentially expressed genes (significantly up- or down-regulated) in Ring stages of the 3D7+PfAlba2-Ty1 strain

Supplementary Table S4: List of differentially expressed genes (significantly up- or down-regulated) in Trophozoite stages of the 3D7+PfAlba2-Ty1 strain

Supplementary Table S5: List of differentially expressed genes (significantly up- or down-regulated) in Ring stages of the 3D7+PfAlba3-Ty1 strain

Supplementary Table S6: List of differentially expressed genes (significantly up- or down-regulated) in Trophozoite stages of the 3D7+PfAlba3-Ty1 strain

Supplementary Table S7: List of differentially expressed genes (significantly up- or down-regulated) in Ring stages of the 3D7+PfAlba4-Ty1 strain

Supplementary Table S8: List of differentially expressed genes (significantly up- or down-regulated) in Trophozoite stages of the 3D7+PfAlba4-Ty1 strain

Supplementary Table S1: **Mapping statistics and SRA accession IDs of the fastq files generated in this study**

Replicate	Strain	Sample Name	FASTQ reads	Trimmed FASTQ Reads	Number of uniquely mapped reads (Q20)	% mapping	Genomic coverage	SRA accession ID
1	3D7	3D7_T1	17985498	17643833	16769911	95.05%	53.90442736	SRR28384927
	3D7+empty vector	pLN_R1	26498909	26007851	21643763	83.22%	69.57071212	SRR28384925
		pLN_T1	18361044	17989234	15771501	87.67%	50.69518437	SRR28384923
	3D7+PfAlba4-Ty1	A4_R1	18894449	18399258	13009300	70.71%	41.81649306	SRR28384915
		A4_T1	19808303	18370992	16682052	90.81%	53.62201745	SRR28384930
	3D7+PfAlba2-Ty1	A2_R1	26468269	26130572	19310703	73.90%	62.07143181	SRR28384932
		A2_T1	13731273	13553440	11849850	87.43%	38.08961053	SRR28384921
	3D7+PfAlba3-Ty1	A3_R1	26960471	26212607	20314305	77.50%	65.29736373	SRR28384919
		A3_T1	36017961	34349876	28164787	81.99%	90.53159048	SRR28384917
2	3D7	3D7_R2	18221214	17773377	14016752	78.86%	45.05480023	SRR28384928
		3D7_T2	28269818	27846381	25914487	93.06%	83.29833009	SRR28384926
	3D7+empty vector	pLN_R2	29318659	28950413	24230560	83.70%	77.88559292	SRR28384924
		pLN_T2	31451701	28163574	26011675	92.36%	83.61072671	SRR28384922
	3D7+PfAlba4-Ty1	A4_R2	15475399	15111357	12142728	80.35%	39.03102404	SRR28384914
		A4_T2	23545847	23157744	21906919	94.60%	70.41658861	SRR28384929
	3D7+PfAlba2-Ty1	A2_R2	22904881	22593287	17736782	78.50%	57.01229285	SRR28384931
		A2_T2	29145905	28670756	25039762	87.34%	80.48665445	SRR28384920
	3D7+PfAlba3-Ty1	A3_R2	50120983	49078208	41921998	85.42%	134.7521341	SRR28384918
		A3_T2	37591001	37074004	33768167	91.08%	108.5428363	SRR28384916

R=Ring; T=Trophozoite

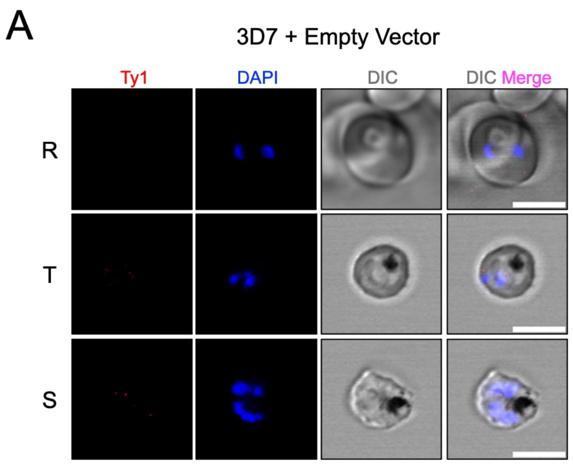

B

Strain	Stage	Manders' Coefficients* using thresholds		Pearson's Coefficient
		Fraction A overlapping with B	Fraction B overlapping with A	
3D7 + PfAlba2-Ty1	R	0.478	0.54	0.558
	T	0.103	0.572	0.304
	S	0.306	0.702	0.443
3D7 + PfAlba3-Ty1	R	0.584	0.624	0.46
	T	0.433	0.989	0.658
	S	0.584	0.624	0.46
3D7 + PfAlba4-Ty1	R	1.000	0.492	0.756
	T	0.564	0.735	0.654
	S	0.823	0.562	0.671

*A = Ty1 Red; B = DAPI Blue

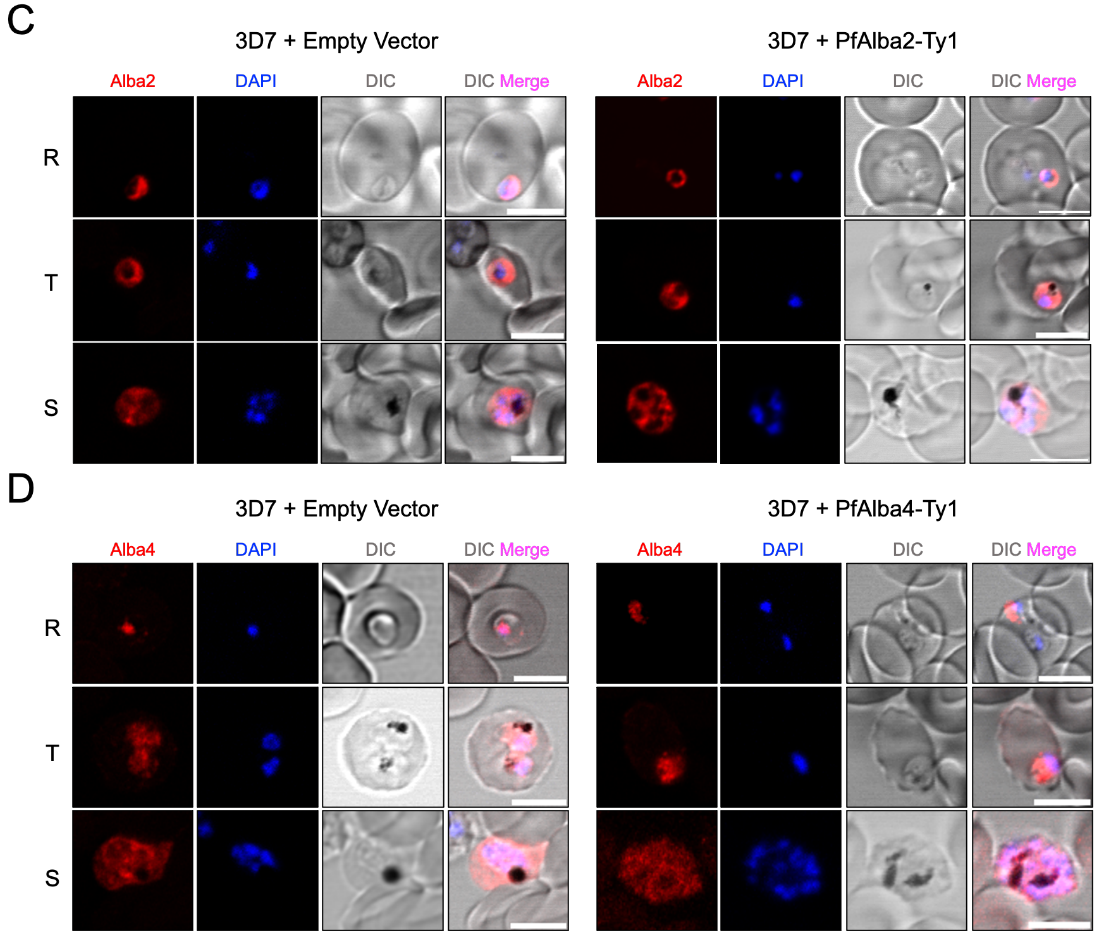

Supplementary Figure S1: Immunofluorescence assays (IFAs). (A) IFAs of 3D7+empty vector parasites stained with anti-Ty1 antibodies. Antibodies used included rabbit anti-Ty1 (red). Nuclei were labelled with DAPI (blue). Scale bar represents 5 µm. **(B)** Colocalisation of the anti-Ty1 and DAPI signals in Figure 1D was evaluated by calculating the Manders' and Pearson Correlation Coefficients in ImageJ. **(C)** IFAs were used to determine the staining pattern of rabbit anti-PfAlba2 antibodies (red) in the 3D7+empty vector and 3D7+PfAlba2-Ty1 parasites. Nuclei were labelled with DAPI (blue). Scale bar represents 5 µm. **(D)** IFAs

were used to determine the staining pattern of rabbit anti-PfAlba4 antibodies (red) in the 3D7+empty vector and 3D7+PfAlba4-Ty1 parasites. Nuclei were labelled with DAPI (blue). Scale bar represents 5 μm. For all parts, R = Ring; T = trophozoite; S = schizont. All of the cultures used for IFAs contained 5 μg/ml of blasticidin-S.

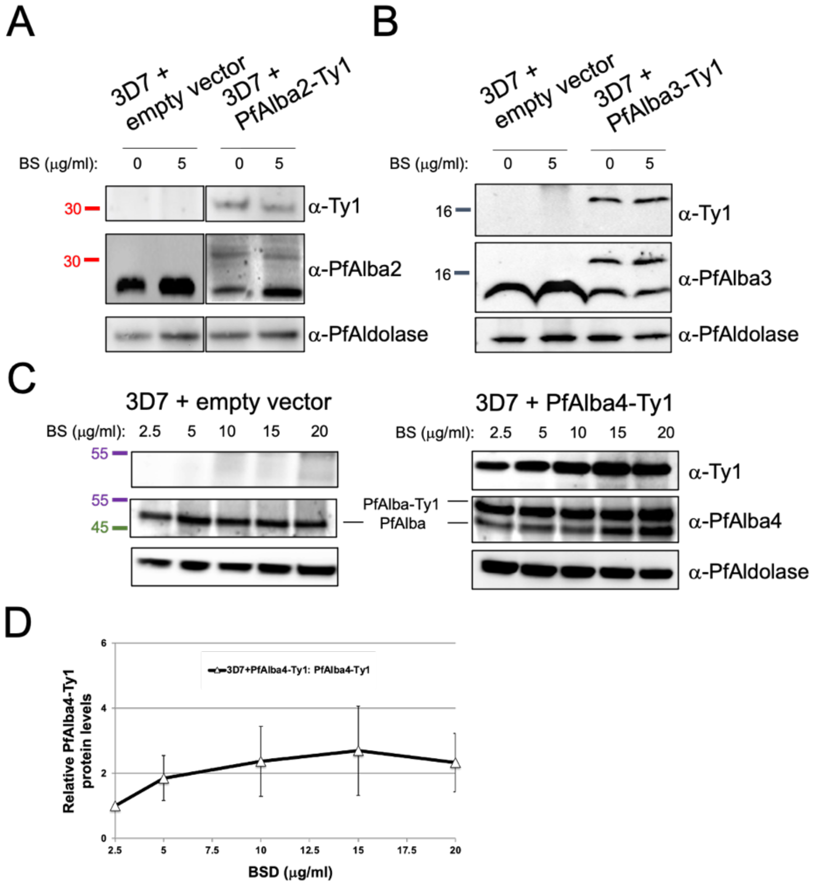

Supplementary Figure S2: Blasticidin dose response of PfAlba-Ty1 expression. (A) & (B) Denaturing gel electrophoresis was used to compare protein lysates from 3D7+empty vector to lysates from **(A)** 3D7+PfAlba2-Ty1 or **(B)** 3D7+PfAlba3-Ty1 transfectants grown in the presence of 0 or 5 μg/ml of blasticidin-S (BS). Western blotting was performed using rabbit anti-Ty1 or anti-PfAlba antibodies. PfAldolase was used as a loading control. **(C)** Denaturing gel electrophoresis was used to separate protein lysates from 3D7+empty vector or 3D7+PfAlba4-Ty1 transfectants grown in the presence of increasing concentrations of blasticidin-S (BS; 2.5–20 μg/ml). Western blotting was performed using mouse anti-Ty1 or anti-PfAlba4 antibodies. PfAldolase was used as a loading control. **(D)** Densitometry analysis was used to quantify the amounts of PfAlba4-Ty1 in part C. Data were normalized to the 2.5 μg/ml sample for each BS concentration, followed by the normalization of the PfAlba4-Ty1 signal to the PfAldolase signal. The data are the means ± standard error (S.E.; error bars) of at least three independent studies.

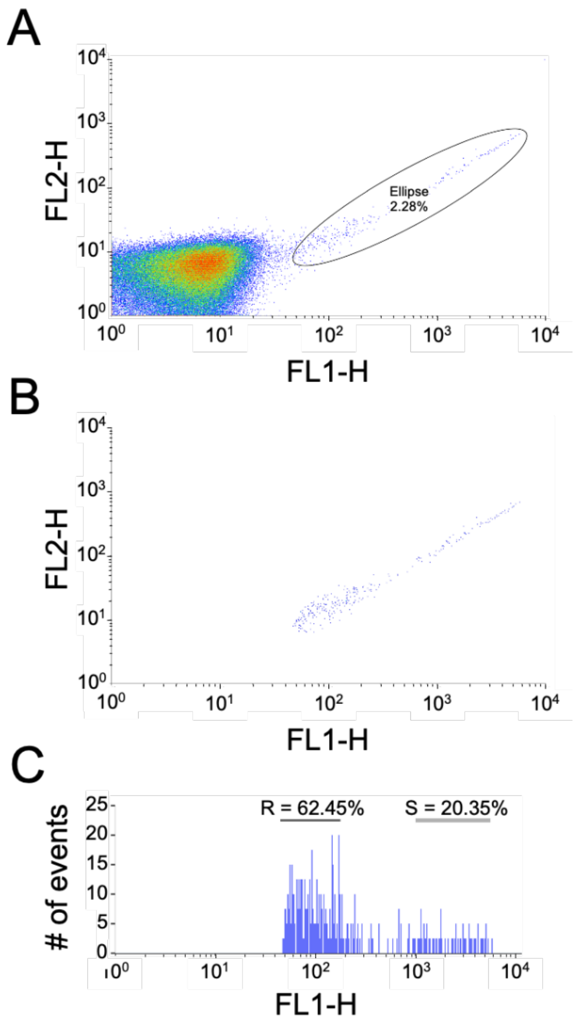

Supplementary Figure S3: Sample flow cytometry plot corresponding to Fig. 2B. Flow cytometry analysis was performed to quantify the numbers of rings, trophozoites (T) and schizonts in a given *P. falciparum* culture. **(A)** Plot of FL1-H (green) and FL2-H (yellow) are shown, which correspond to SYBR Green I staining of DNA. An ellipse was used to gate infected RBCs, which are independently shown in **(B)**. **(C)** A histogram was used to count the number and percentage of rings (R) and schizonts (S) in the culture.

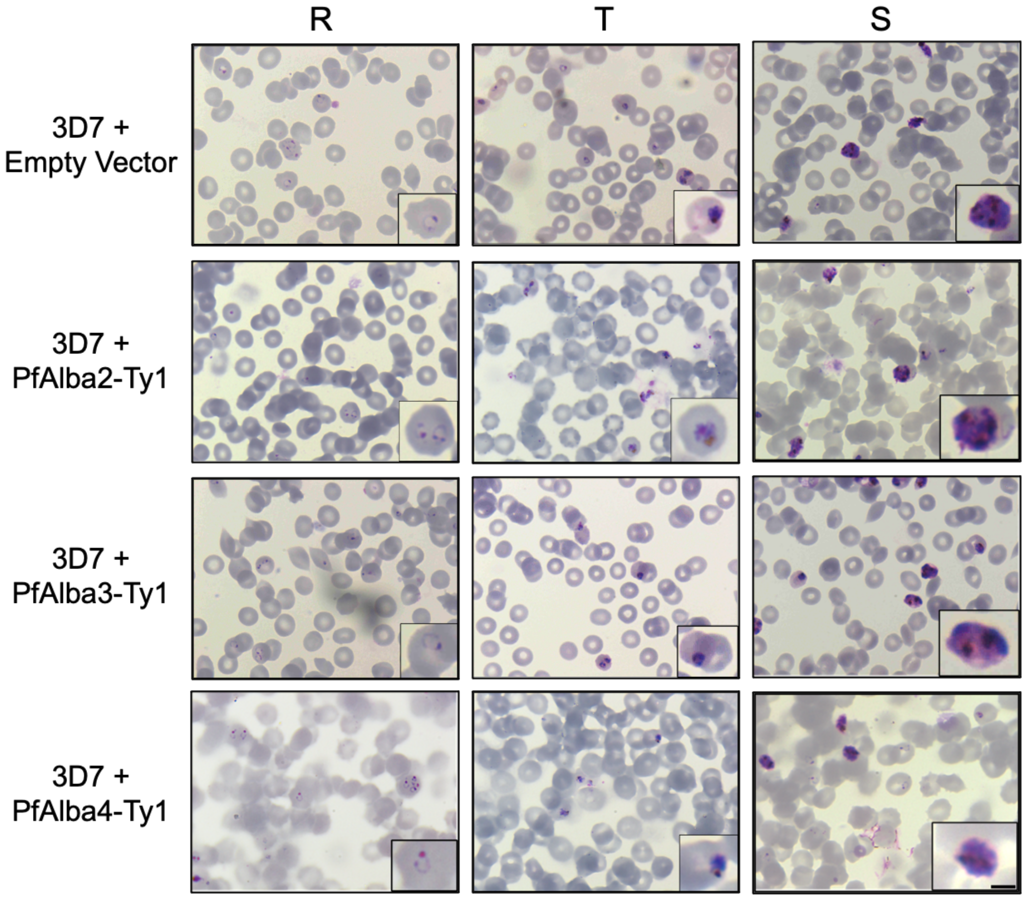

Supplementary Figure S4: Giemsa staining of blood smears of different stages of the PfAlba-Ty1 transfectant cultures. R = Ring; T = trophozoite; S = schizont. All of the cultures used for Giemsa staining contained 5 µg/ml of blasticidin-S. Scale bar represents 10 µm.

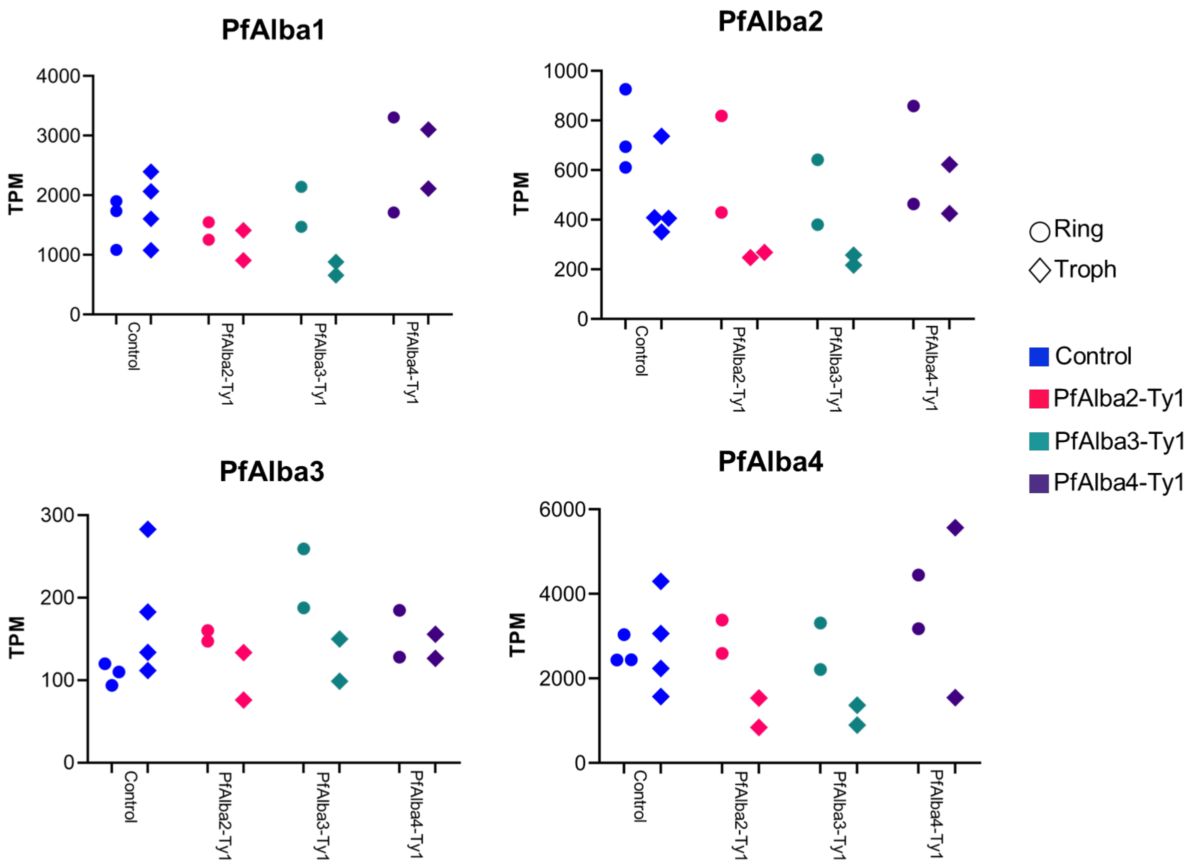

Supplementary Figure S5: Analysis of PfAlba gene expression in the overexpression and control parasite strains. Transcripts per million (TPM)-normalised expression values of PfAlba1, PfAlba2, PfAlba3, and PfAlba4 were calculated for the ring and trophozoite RNA-seq data of the three overexpression strains and compared to controls. GraphPad Prism was used to plot the data.

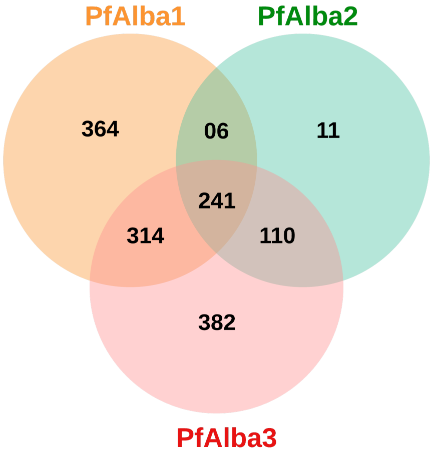

Supplementary Figure S6: Comparative analysis of genes that are differentially expressed in the 3D7+PfAlba2-Ty1 and 3D7+PfAlba3-Ty1 transfectants relative to 3D7+PfAlba1-Ty1. A Venn diagram was used to represent overlapping transcripts between the three overexpression strains during trophozoite stages. Data for 3D7+PfAlba1-Ty1 were obtained from Vembar *et al.,* 2015 (https://genomebiology.biomedcentral.com/articles/10.1186/s13059-015-0771-5).

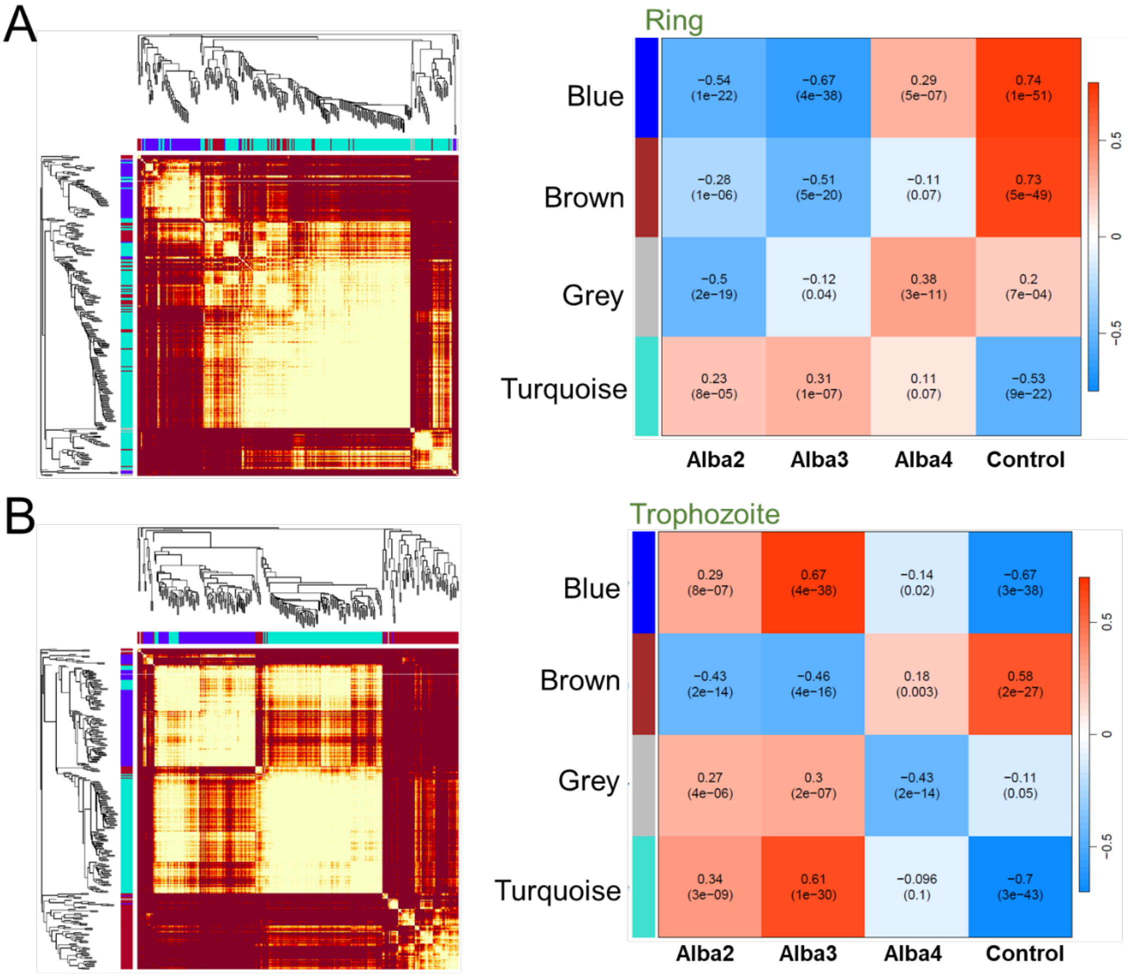

Supplementary Figure S7: Weighted gene co-expression network analysis of the transcriptomes of the PfAlba transfectant parasites. (A & B) *Left panel:* Heatmap of topological overlap in the gene network of all **(A)** ring and **(B)** trophozoite stage transcriptomes generated in this study. In the heatmap, each row and column correspond to a gene; light yellow represents lower topological overlap, while progressively darker red indicates higher overlap. Dark squares along the diagonal correspond to modules. The gene dendrogram and module assignment are shown along the left and top. *Right panel:* Module-trait relationship heatmap for all **(A)** ring and **(B)** trophozoite stage transcriptomes. Each row corresponds to a module and each column corresponds to a trait, *i.e.,* sample type: 3D7+PfAlba2-Ty1 (Alba2), 3D7+PfAlba3-Ty1 (Alba3), 3D7+PfAlba4-Ty1 (Alba4), and controls. The colour legend illustrates the degree of correlation between a given module and trait with significance of the correlation represented as p-values within parantheses.

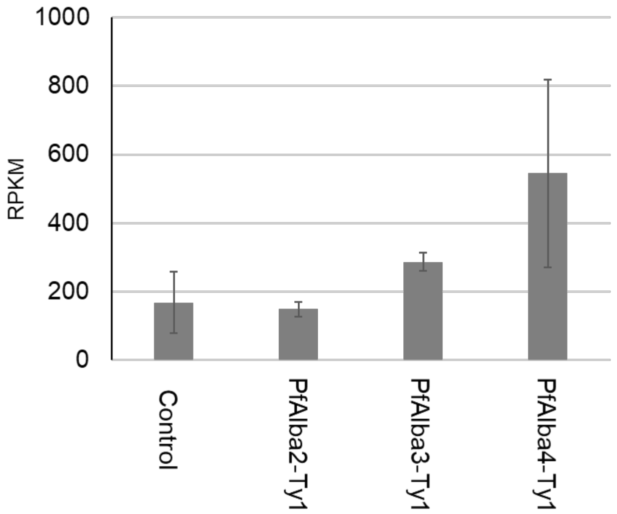

Supplementary Figure S8: Expression levels of the RUF6 ncRNA family in the PfAlba overexpression strains. Reads per kilobase of transcript per million (RPKM) values of 15 RUF6 genes were extracted from ring stage RNA-seq data for the indicated strain and their sum calculated. The graph was plotted using Microsoft Excel.

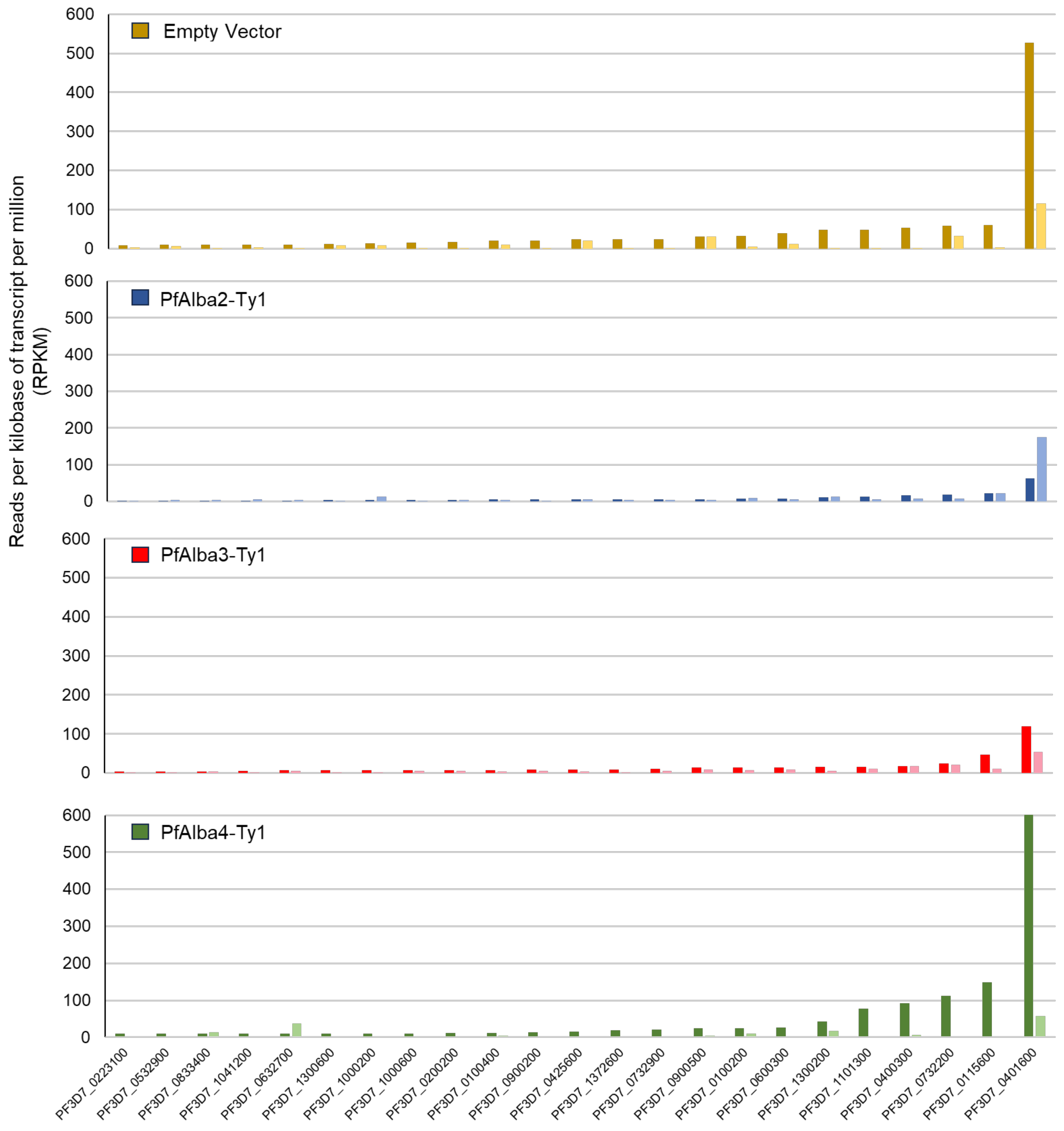

Supplementary Figure S9: Effect of excess PfAlba protein levels on *rifin* gene expression. Reads per kilobase of transcript per million (RPKM) values of 23 *rifin* genes that were found to be highly expressed in the trophozoite stage transcriptomes generated in this study were plotted.
